# Mechanistic analysis of a synthetic inhibitor of the *Pseudomonas aeruginosa* LasI quorum-sensing signal synthase

**DOI:** 10.1038/srep16569

**Published:** 2015-11-23

**Authors:** O. Lidor, A. Al-Quntar, E. C. Pesci, D. Steinberg

**Affiliations:** 1 Biofilm Research Laboratory, Institute of Dental Sciences, Faculty of Dental Medicine, The Hebrew University of Jerusalem, Jerusalem, Israel; 2 Institute of Drug Research, School of Pharmacy, The Hebrew University of Jerusalem, Jerusalem, Israel; 3 Department of Microbiology and Immunology, Brody School of Medicine, East Carolina University, USA

## Abstract

*Pseudomonas aeruginosa* is an opportunistic Gram-negative pathogen responsible for many human infections. LasI is an acyl-homoserine lactone synthase that produces a quorum-sensing (QS) signal that positively regulates numerous *P. aeruginosa* virulence determinants. The inhibition of the LasI protein is therefore an attractive drug target. In this study, a novel *in silico* to *in vitro* complementation was applied to screen thiazolidinedione-type compounds for their ability to inhibit biofilm formation at concentrations not affecting bacterial growth. The compound (*z*)-5-octylidenethiazolidine-2, 4-dione (TZD-C8) was a strong inhibitor of biofilm formation and chosen for further study. Structural exploration of *in silico* docking predicted that the compound had high affinity for the LasI activity pocket. The TZD-C8 compound was also predicted to create hydrogen bonds with residues Arg30 and Ile107. Site-directed mutagenesis (SDM) of these two sites demonstrated that TZD-C8 inhibition was abolished in the *lasI* double mutant PAO-R30D, I107S. In addition, *in vitro* swarming motility and quorum sensing signal production were affected by TZD-C 8, confirming this compound alters the cell to cell signalling circuitry. Overall, this novel inhibitor of *P. aeruginosa* quorum sensing shows great promise and validates our mechanistic approach to discovering inhibitors of LuxI-type acyl-homoserine lactone synthases.

*Pseudomonas aeruginosa* is a persistent opportunistic pathogen that poses a significant health risk to hospitalized patients and immune-deficient patients, such as those with cystic fibrosis^1^. This Gram-negative bacterium thrives in nosocomial conditions by utilizing numerous virulence factors, some of which are controlled via quorum-sensing (QS) systems. Quorum sensing is a mechanism by which bacteria monitor and respond to their own population density. Quorum sensing in *P. aeruginosa* is an essential tool for adaptation to environmental conditions such as oxidative stress^2^ and nutrient deprivation^3^. The quorum-sensing response usually involves activating numerous physiological pathways, including those responsible for biofilm formation^2^,^4^.

A biofilm is a self-constructed three-dimensional community of bacteria. Biofilm formation enables bacteria to live on or in many different substrates. *P. aeruginosa* biofilms attach strongly to a surface by producing an extracellular matrix using compounds such as exo-polysaccharides (EPS) or DNA^4^. This creates a physical barrier that serves as effective protection against host immune cells. However, this barrier is ineffective in blocking small molecules^5^. Therefore, the use of small molecules is a potential anti-biofilm strategy.

The quorum-sensing mechanism in *P. aeruginosa* is mediated by cell-permeable acyl-homoserine lactone (HSL) signals. Accumulation of the intercellular signals C4-HSL and 3-oxo-C12-HSL activates the transcriptional regulators LasR and RhlR, respectively, through the specific binding of each signal. These response regulators increase the expression of many genes in the bacterial genome, resulting in a swarming motility phenotype^6^, biofilm formation^4^ and the expression of numerous other virulence genes, such as *lasB*, *lasA*, *apr* and *toxA*^7^,^8^,^9^.

In *P. aeruginosa*, the compound 2-heptyl-3-hydroxy-4-quinolone (Pseudomonas Quinolone Signal, PQS)^10^,^11^ is a third intercellular signal. PQS serves as a link between the *las* quorum-sensing system and the *rhl* quorum-sensing system and seems to elicit its effects on virulence through the *rhl* quorum-sensing system^9^,^11^,^12^. The *pqsABCDE* operon produces many quinolone secondary metabolites, some of which are responsible for iron acquisition from the environment^10^,^13^. PQS has recently been linked to biofilm formation and integrity, and the anti-oxidative properties of biofilms^2^,^4^.

*P. aeruginosa* HSLs are produced by the HSL-synthases LasI and RhlI from *S*-adenosyl-*L*-methionine (SAM) and acyl-acyl-carrier protein (acyl-ACP) substrates^14^. LasI is similar to other synthases, such as LuxI, TofI and EsaI. These HSL-synthases possess an overall structure that includes a hydrophobic binding pocket for acyl-chain binding^14^. A prior study of LasI structure and function^14^ revealed a crucial V-shaped substrate-binding cleft. This cleft accommodates the substrates 3-oxo-C12-acyl carrier protein (Acyl-ACP) and SAM. The V-shaped substrate-binding cleft in the LasI protein is a conserved binding pocket that is crucial for HSL production. HSL production is enabled by a specific interaction between the conserved N-terminal residues Arg23, Phe27 and Trp33 and a wide cleft that facilitates the binding of the acyl-phosphopantetheine chain of acyl-ACP^14^.

Thiazolidinedione (TZD)-type molecules were previously shown to have a bactericidal effect on gram-positive bacteria such as *Streptococcus pneumoniae*^15^ and an anti-QS effect on *Vibrio harveyi*^16^. TZD was also found to have an effect on *Candida albicans* biofilms^17^, anti-adhesion activity and cell morphology^18^. The chemistry of TZD molecules has been reviewed extensively^19^. Taken together, the available preliminary data led us to hypothesize that TZD could target the quorum sensing systems of *P. aeruginosa* to negatively affect biofilm formation. Therefore, we explored the anti-quorum-sensing effect of TZD derivatives on *P. aeruginosa*, which led to the discovery of a novel inhibitory compound and the proposal of its potential mechanism of action.

## Materials and Methods

### Synthesis of TZDs

Synthesis of C8-10 TZD molecules was performed according to a previously described method^17^,^18^,^19^. Briefly, 0.117 g 1 mmol of thiazolidine-2, 4-dione in 8 mL of ethanol was added to 0.085 g 1 mmol of piperidine. This was followed by the addition of 0.115 g (0.9 mmol) of octanal. The mixture was stirred for 30 min at room temperature, and then refluxed in ethanol for 24 hours. After cooling the mixture to 0 °C, 5 ml of 1 M HCl solution was added. The mixture was then was maintained in the refrigerator for a few days. Following this incubation, the precipitate was filtered, washed with petroleum ether, dried and analysed by NMR. Melting point and elemental analyses were also performed to give TZD-C8 with a 60% yield and a chemical purity above 95%. The synthesis of TZD-C10 was similar to the synthesis of TZD-C8, except for the addition of 0.141 g (0.9 mmol) of decanal in place of octanal. TZD synthesis is described in Figure S1. The structure and purity of synthetic molecules were verified via ^1^H-NMR and ^13^C-NMR (Supplementary Data Figure S2).

### Bacterial Strains, Plasmids and Growth Conditions

The bacterial strains and plasmids used are listed in Table S1. *P. aeruginosa* strain PAO1 was stored at −70 °C in 10% (w/v) skim milk media (NEOGEN, Lansing, Michigan, USA). *P. aeruginosa* cultures were grown in Lennox L. agar (LB) at 37 °C and 5% CO_2_ with vigorous shaking. To maintain plasmids, carbenicillin or tetracycline was added to the growth media in concentrations of 200 μg/ml or 30 μg/ml, respectively.

The plasmid pJP1-comp was constructed as follows. Two primers, 5′-ATGATCGTACAAATTGGTCGGC-3′, 5′-AAAAAAGCTTTTTACAGCGGATTCGGCA-3′, were used to amplify the *lasI* gene region in a polymerase chain reaction (PCR) reaction with *PfuUltra* DNA polymerase (Agilent®, Santa Clara, California, USA). The first primer hybridized to a DNA region centered at the translational start codon of *lasI*. The second primer hybridized to a DNA region centered at the *lasI* stop codon and contained an additional *HindΙΙΙ* restriction site at the 5′-terminus (with an additional 5A nucleotides to aid digestion). The amplified fragment was digested with *HindΙΙΙ* and ligated into pEX1.8. Vector DNA was digested with *EcoRI*, treated with Klenow fragments, and finally digested with *HindIII*. The resulting recombinant plasmid (pJF1-comp), which contained the *lasI* gene, was electroporated into *P. aeruginosa* strains to conduct *lasI* gene complementation according to a method previously reported in the literature^20^.

### Static Biofilm Assay

A static biofilm growth system was conducted in 96-well plates (Nunclon®, Roskilde, Denmark). Bacterial suspensions with absorbance measurements at 660 nm (A_660_) of between 0.05 and 0.13 were incubated in LB media for 24 hours at 37 °C and 5% CO_2_ with vigorous shaking. The biofilm was subjected to two washes with 0.9% saline. Biofilms located at the bottom of the micro-wells were analysed using an Olympus confocal laser scanning microscope (CLSM) with 10 × lenses and 488/510 and 545/610 nm excitation/emission filters. Signals were produced by bacteria harbouring the pMRP9-1 plasmid. GFP produced and localized to live cells was also detected, similar as in previous studies^2^,^21^. All signals were calculated using Olympus FLUOVIEW FV300 application software (Tokyo, Japan). The biofilm formed on the sides of microwells at the liquid-air interface and this region was specifically analysed using crystal violet as a control (Supplementary Data Figure S3).

### Swarming

The effect of TZD on the swarming phenotype of *P. aeruginosa* was tested using the method described by Tremblay and Deziel^22^ and 0.5% modified M9 agar plates. Agar swarm plates were subjected to 60 min of air drying prior to inoculation with 5 μl of a bacterial suspension with an A_660_ of 3.0. The plates were incubated for 24 hours at 30 °C. Following incubation, images of the agar swarm plates were collected using a Canon® G5 5.0 megapixel camera with a 7.2–28.8 mm lens. Images were analysed using Image-Pro Plus 7.0 software (Media Cybernetics, Rockville, Maryland, USA). All swarming sizes were normalized according to plate size to calibrate the calculated area of swarming (in units of pixels/cm^2^).

### Modified β-Gal Plate Assays for *E. coli*: HSL production assay

To test the effects of TZD derivatives on the activity of RhlI and LasI, culture supernatants from tested *P. aeruginosa* strains grown in LB medium for 4–8 hours at 37 °C and 5% CO_2_ with vigorous shaking were extracted with ethyl acetate as described elsewhere^9^,^23^.

The amount of C4-HSL and C12-HSL in the extracts was determined using an *E. coli* bioassay strain carrying both the *lasB-lacZ* translational fusion and *tacp-lasR* under the control of the *lac* promoter (for detecting C12-HSL)^23^,^24^ or *tacp-rhlR* and the *rhlA*′*-lacZ* reporter gene fusion (for detecting C4-HSL)^24^.

To measure β-Gal production from the reporter fusions, a bacterial suspension of the bioassay strain was diluted in LB agar media supplemented with a final concentration of 80 μg/ml of 5-bromo-4-chloro-3-indolyl-β-D-galactopyranoside (X-gal) at a ratio of 1:10. The mixture was allowed to solidify at room temperature. A 7 mm Oxoid^TM^ Blank susceptibility disks (Thermo Scientific, MA, USA) that had absorbed 5 μl of a bacterial extract were carefully placed on top of dried 25 ml LB agar plates and incubated overnight at 30 °C to allow a blue colour to develop (based on a protocol described by Golberg *et al.*^25^). The blue zone was analysed by cutting it from the agar plate and placing it into 1 ml of silica “glass milk” solution (MP, Solon, OH, USA) for 10 min at 55 °C to fully dissolve the agar. The absorbance at A_650_ was then determined using a GeneQuant 1300 spectrophotometer (GE Healthcare, Uppsala, Sweden). β-Gal background activity was determined using a non-exposed reporting strain as a negative control and this value was subtracted from the experimental assays. Calibration curves of the bioassay were constructed in response to exogenous synthetic 3-oxo-C12-HSL (modified b-Gal plate assay sup. data) as well as C4-HSL reporting (Supplementary Data Figure S4).

### PQS Quantification

To test the effect of TZD-C8 on PQS production, *P. aeruginosa* cultures were extracted with acidified ethyl acetate. Extractions were performed using cultures with an absorbance value of 1.0 at A_595._ Extracts were dried, re-suspended in 1:1 ethyl acetate-acetonitrile, and separated by thin-layer chromatography (TLC), as previously described^26^,^27^. The extract was spotted onto the TLC plate as follows: 7 μl of bacterial extract or 5 μl of 20 μM PQS synthetic standard (Sigma) and PQS quantity was then determined by exposing resolved TLC plates to 325 nm UV light.

### RNA Extraction

The extraction of total RNA was performed as previously described^28^ with slight modifications. Total RNA was extracted from planktonic log-phase bacterial cultures using the RNeasy Mini Kit (Qiagen, Hilden, Germany) according to the manufacturer’s instructions (Protocols 5 and 7 of the RNAprotect bacteria reagent handbook), including a non-column DNA removal treatment (Appendix B of the RNAprotect handbook). Briefly, planktonic *P. aeruginosa* cells were suspended in RNAprotect reagent (Qiagen, Hilden, Germany) and placed into a 2 ml microcentrifuge tube containing 0.4 ml of 1 mm diameter glass beads (Sigma-Aldrich, St. Louis, MO, USA). The cells were disrupted with the aid of a FastPrep-24 homogenizer cell disrupter (MP Biomedicals, Santa Ana, California, USA). Further extraction was performed using RNeasy Mini Kit protocols. The RNA concentration was determined spectrophotometrically using a Nanodrop instrument (ND-1000; Nanodrop Technologies). The integrity of the RNA samples was assessed by agarose gel electrophoresis using a 2100 Bioanalyzer Instrument (Agilent, Santa Clara, California, USA), data is described elsewhere (Supplementary Data, Bioanalyzer data).

### Microarray

An RNA expression microarray was used to compare the gene expression patterns of *P. aeruginosa* strain PAO1 grown in the presence or absence of TZD-C8. The microarray utilized fully printed arrays of the bacterial genome attached to custom-made DNA microchips (TIGR) as previously described^28^. An RNA protector/stabilizer was added, and bacteria were then mechanically disrupted using a FastPrep-24 homogenizer (Qiagen). RNA extraction and purification was completed using the Qiagen RNeasy Mini Kit prior to cDNA production. Probe labelling was performed using 20 μg of total RNA, random hexamer primers (3 μg/μl, 3 μl) and RNase inhibitor (5 units/μl, 1 μl) according to guidelines described elsewhere^29^. Following incubation at 70 °C for 10 min, the reaction mix was chilled on ice for 10 min. The primer-RNA solution was then added to the reverse transcriptase mix along with 0.6 μl of 10 mM aminoallyl-dUTP, 3 μl of 0.1 M DTT and 2 μl of Superscript 2 reverse transcriptase (Invitrogen/Life Technologies). The reaction mixture was incubated at 42 °C for 2 h. The reaction was terminated by adding 10 μl of 0.5 M EDTA. The RNA was hydrolysed with 10 μl of 1 M NaOH at 65 °C for 30 min. MinElute PCR purification kits were used to purify cDNA. Next, 3 μl of 1 M sodium bicarbonate (pH 9.3) was added to the cDNA solution. The solution was mixed via pipetting, and the tubes were incubated at room temperature for 1 h with horizontal shaking.

The array scanning analysis was performed as follows. Arrays were read as described previously^28^,^30^ with a GenePix 4000B scanner (Axon, Foster City, CA) at 10 μm resolution and variable photomultiplier tube (PMT) voltage settings to obtain maximum signal intensities with <1% (wt/vol) probe saturation. The resulting images were analysed by determining the fluorescence intensity of the two dyes at each spot. Eight separate experiments were conducted to obtain statistically significant results.

Microarray data are available in the ArrayExpress database (www.ebi.ac.uk/arrayexpress) under accession number E-MTAB-3144.

### *In silico* Analysis

Ligand molecules designed for docking were constructed using ACD/lab Chemsketch freeware^31^. Files were converted from 2D and 3D structures to .pdb using OpenBabel software^32^. The center of the active site was coordinated between the ligand and the protein using the AutoDock program^33^. Autodock was also used to prepare the .pdb files used for docking; such actions included the addition of polar hydrogen atoms and Gasteiger charges. The size of the grid box in AutoDock Vina was kept at the default settings of 30, 30, and 30 for X, Y, and Z, respectively. Vina was implemented through a shell script provided by the Vina developers. The binding affinity of the ligand was observed as a negative score with units expressed as kcal/mol. AutoDock Vina generated nine poses of the ligand with distinguishable binding energy for each ligand input. Docking in Vina is done in a similar fashion for the following three types of ligand: TZD, TZD-C8 and TZD-C10. Docking was performed over rigid protein structures of PqsR, QscR, LasR and LasI. The residues involved in ligand docking between LasI and the TZD-C8 molecule were verified using PDBsum analysis^34^ of the .pdb docking files (PDB case no. k032, i772 and k031, Supplementary Data). This site was selected for virtual screening. The .pdb files containing poses with the highest Vina affinity scores were compared using LIGPLOT. In addition, the stereochemical quality of each .pdb file was verified using PROCHECK, and a corresponding protein interaction interface analysis was performed using PISA^35^, results are in Supplementary Data.

### Site-Directed Mutagenesis (SDM)

SDM was conducted using a PCR-based method^36^. We used the QuickChange Lightning kit (Stratagene, La Jolla, CA, USA), a set of *lasI*-specific primers (Table S3) and the plasmid pJP1-comp as a template (Table S1). The SDM reaction created single and double amino acid substitutions at Arg30 and/or Ile107 in the LasI protein to examine the viability of the hydrogen bonds predicted by PDBsum. All primers were designed for SDM using the Agilent primer design program to achieve optimal melting points for the substitution of bases by lowering the free energy cost. All mutations were verified by sequencing using the ABI PRISM 3730xl DNA Analyser [Applied Biosystems (Supplementary Data)].

### Statistical analysis

All Variables analysed in this study were quantitative. Descriptive statistics includes mean and SD as well as median and range. For variables for which the sample size was small (N < = 12) the Mann-Whitney non-parametric test was applied for comparing two independent groups. The comparison of three independent groups was done using the non-parametric Kruskal-Wallis test, the Mann-Whitney test for pairwise comparisons, and the Bonferroni correction of the significance level. These tests were applied according to the Shapiro Wilk test, the data were not normally distributed. All tests applied were two-tailed, and a p-value of 5% or less was considered statistically significant. Analyses were performed using SPSS software, version 21.

## Results

### TZD negatively affects biofilm formation formation

**Figure 1:**
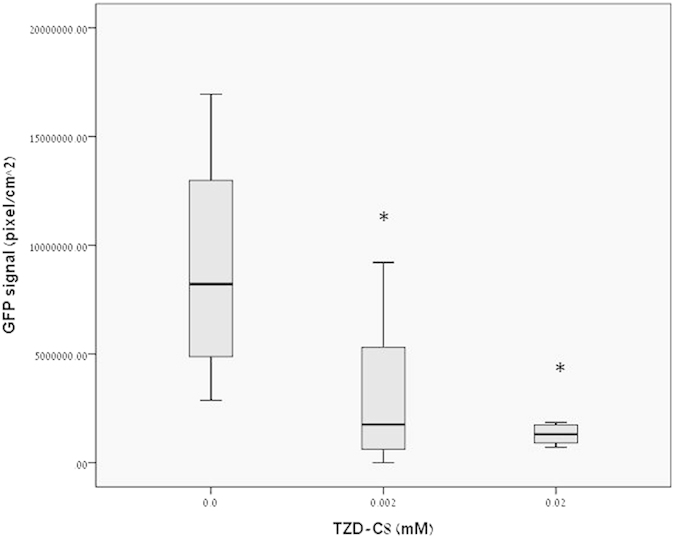
Analysis of 24 h biofilms. Static biofilm analyzed under confocal microscopy, detecting GFP produced from strain PAO1 carrying pMRP9-1 exposed to 0, 0.002, and 0.02 mM concentrations of TZD-C8, every bar represents N > 20 of biofilm sections. *Statistical comparison of three independent groups was done using the non-parametric Kruskal-Wallis test, with significant statistical difference between control and selected bar with P < 0.05, T-test were considered as non-equal variance groups, statistical deviations (S.D.) are included.

The effect of sub-lethal doses of TZD derivative of TZD-C8 (Fig. 1) on surface biofilm formation of *P. aeruginosa* on the bottom of microwell plate was analysed. We performed a phenotypic analysis that primarily screened for biofilm formation after exposure to TZD derivatives. Biofilm formation was reduced, as measured by GFP fluorescence in live cells. This reduction occurred in a dose-dependent manner, as concentrations of TZD-C8 increased in a static model. Exposed biofilms exhibited lower GFP intensity levels, indicating that fewer cells were detected in the plate biofilm model. Biomass decreased up to 70% after exposure to 20 μM TZD-C8. [Note: the minimal concentration of TZD-C8 that affects the growth of bacteria is 20 mM (Supplementary Data, Figure S5)]

### TZD effects on gene expression in *P. aeruginosa*

The effect of TZD-C8 on gene expression was determined by microarray analysis of the wild-type strain PAO1. The gene expression profiles of bacteria in a planktonic state in the presence or absence of 0.02 mM TZD-C8 were compared. *P. aeruginosa* strain PAO1 exposed to TZD-C8 exhibited differential expression of 51 genes (Table S2). Most interestingly, the genes of the *pqsABCDE* operon were downregulated, with fold changes of −0.78, −0.9, −1.08, −0.79 and −0.95 observed for *pqsA*, *pqsB*, *pqsC*, *pqsD and pqsE*, respectively. The expression of *lasI* was downregulated; a fold change of −0.47 was observed. These results indicate that TZD-C8 interferes with both PQS and 3-oxo-C12-HSL signalling pathways. Another interesting regulation change was the increased expression of the efflux channel protein operon *mexAB-oprM*, which had fold changes of 1.22, 1.22 and 1.00. Expression of these proteins is induced constitutively in wild-type *P. aeruginosa* as they function in antibiotic resistance^37^. A downregulation in the arginine transporting system operon *arcDABC* was noted, with fold changes of −2.28, −2.04, −1.98 and −2.08 observed for *arcA*, *arcB*, *arcC and arcD*, respectively. This could be attributed to the ANR/DNR downregulation process^38^. ANR/DNR downregulation additionally affects the *ccpR*, *azu*, *aer* and *clpB* genes^38^, as depicted in our microarray results.

### Complementation of the *lasI* mutant

The microarray analysis revealed a negative effect of TZD on the PQS operon. Therefore, we searched for an upstream effect on the *lasI* gene that was responsible for this downregulation of the expression of *pqs* genes.

**Figure 2:**
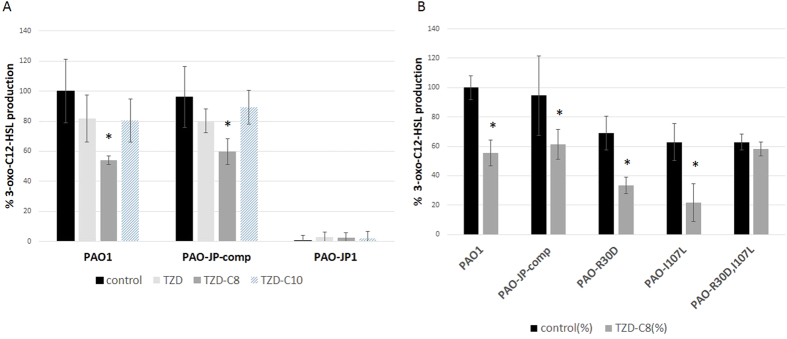
Inhibition of LasI activity by TZD derivatives. Modified β-Gal plate assay to detect HSL using *E.coli* strain MG4 (pKDT17) in the presence of organic extracts of *P. aeruginosa* strains; (**A**) PAO1 (w.t), PAO-JP-comp (∆*lasI* + *lasI* complementation) and PAO-JP1 (∆*lasI*) unexposed Vrs. exposed to 20 μM of TZD molecules: TZD, TZD-C8, TZD-C10. Each bar contains a pool of N = 8 repeats. (**B**) PAO1, PAO-JP-comp, PAO-R30D, PAO-I107S, PAO-R30D, I107S strains unexposed vrs. exposed to 20 μM of TZD-C8. Relative percentage quantification of 3-oxo-C12-HSL was done before and after TZD-C8 (20 μM) exposure. *Statistical significance was analysed by the Mann-Whitney non-parametric test was applied for comparing each two independent groups showing P < 0.05 between selected bar and control ones. Statistical deviations (S.D) are included.

An indirect *in silico* analysis indicated a strong interaction between the LasI protein and the TZD-C8 molecule. This finding led us to perform a direct *in vitro* test for LasI activity. LasI activity was analysed using modified β-Gal plate assays in *E. coli* (Fig. 2A). Extracts from the *P. aeruginosa* wild-type strain PAO1 exposed to TZD-C8 showed 44% and 46% reductions in HSL production (Fig. 2A,B, respectively). Strain PAO1 exposed to TZD-C10 exhibited only a 14% reduction in HSL production. A *lasI*-deletion mutant strain (PAO-JP1) showed minimal HSL production (a null amount of 3-oxo-C12-HSL was produced and detected by this assay, Fig. 2A). A complemented strain, PAO-JP-comp, was created using specific primers (Table S3). PAO-JP-comp showed 33% and 36% reductions in HSL production after exposure to TZD-C8 (Fig. 2A,B, respectively). The complemented strain PAO-JP-comp produced an average of 96% of 3-oxo-C12-HSL (normalized to PAO1) while utilizing the pJP-comp IPTG-induced plasmid. When these cells were exposed to TZD-C10, production was reduced by 11%. These data imply that the derivative compound TZD-C8 specifically interacts with LasI.

*In silico* prediction was used to select mutagenesis targets for the SDM-mutated *lasI* strains (Table S1). All of the SDM-mutated *lasI* strains showed lower *lasR* promoter activity for the 3-oxo-C12-HSL reporter after exposure to 20 μM of TZD-C8 (Fig. 2B). The strain PAO-R30D, which carried an R30D substitution, showed a 35% reduction in HSL production. A 33% reduction in HSL production was observed in the control PAO-JP-comp strain. The strain PAO-I107S contained an I107S substitution that lowered the hydrophobic strength at the binding pocket and this strain exhibited a 41% reduction in HSL production. By contrast, the PAO-R30D, I107S strain (bearing two substitutions, R30D and I107S) showed only a 4% mean reduction in HSL production.

No change in C4-HSL production by strain PAO1 was detected (Figure S4).

### Swarming and PQS signalling affected by TZD-C8

**Figure 3:**
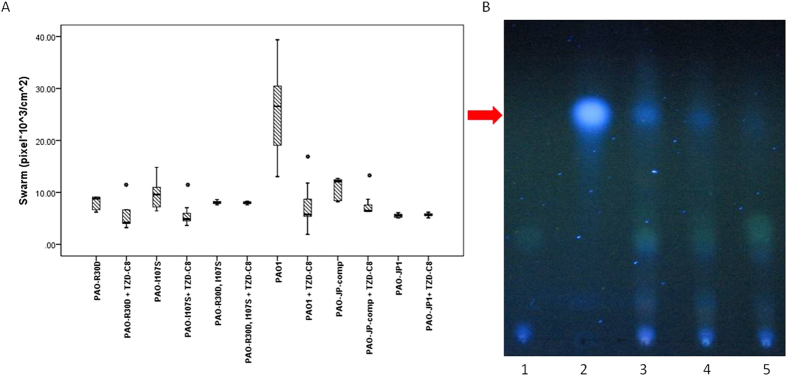
TZD-C8 affects swarming and PQS production. (**A**) Swarming motility area measurement boxplot analysis of *P. aeruginosa* strains (from left to right): strains R30D (PAO-pOL1), I107S (PAO-pOL2), PAO-pOL3, PAO1, PAO-JP-comp, and PAO-JP1—before and after exposure to 0.02 mM TZD-C8 concentration. Each box represents eight different plates. All area measurements were performed by Image-Pro 7.0 software. Results represent N ≥ 5 of swarm plates, in the form of bars plots, *Statistic significance was calculated as two independent Mann-Whitney t-tests between groups showing P < 0.05 between selected bar and control ones. (**B**) PQS production visualized with TLC plate exposed to 325 nm UV light. Lanes: 1- an extract from PQS deficient strain (PAO-QA1), 2–20 μM synthetic PQS (sigma), lanes 3 to 5 are PAO1 strain extracts after exposure to 0, 2 μM and 20 μM of TZD-C8, respectively. The location of the PQS is indicated by a red arrow.

The swarming phenotype of *P. aeruginosa* was also altered by TZD-C8 treatment; this alteration was observed in all strains containing *lasI* (Fig. 3A). Swarming in *P. aeruginosa* was clearly restored after complementing *lasI*-deficient strains. This behaviour change was evident when comparing swarming in PAO-JP1 and the PAO-JP-comp strain. Swarming was most significantly reduced in the PAO1 strain, where it decreased by an average of 72% after exposure to 20 μM TZD-C8. Swarming in PAO-JP-comp was reduced by an average of 40%. No swarming was observed in the PAO-JP1 strain. Our results indicate a great reduction in swarming motility after exposure to 20 μM TZD-C8. This was evident in the mutated strains PAO-R30D and PAO-I107S (33% and 37% mean reductions in swarming, respectively). Swarming motility increased in the double mutant strain by only 4% under the same conditions.

PQS signalling in the PAO1 strain was reduced in a dose-responsive manner after exposure to the TZD-C8 molecule (Fig. 3B). This reduction was first observed with exposure to 2 μM and 20 μM of TZD-C8. A clear decrease in PQS production was seen on the TLC plate at R_f_ ~ 0.6, the same location as the control synthetic PQS (Sigma). No PQS was evident in the control extract taken from the ∆*pqsA* strain (strain PAO-QA1 in Table S1).

### *In silico* evaluation of the interaction between TZD and LasI

In this study, we used the low-molecular-weight compounds TZD, TZD-C8, and TZD-C10 at 114, 230, and 256 g/mol, respectively. Control proteins for the docking screening were selected based on their relevance to QS mechanisms and the analysis was performed using AutoDock Vina. Proteins such as PqsR (pdb file 4JVD) demonstrated low affinity to TZD, TZD-C8 and TZD-C10 with docking calculations in a range from −4.3 to −5.3 kcal/mol (Table S4). Similar calculations were extracted for the interaction between QscR (pdb file 3SZT) and the TZD molecules and affinities were no greater than −6.5 kcal/mol. Of the compounds analysed with LasR (.pdb file 3IX3), the highest affinity was to TZD-C8, at −7.0 kcal/mol. The highest calculated affinity was between LasI and TZD-C8, at −8.1 kcal/mol.

**Figure 4:**
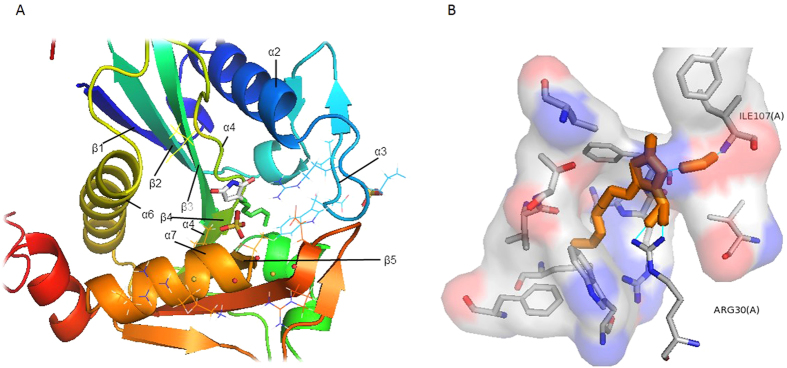
*In silico* Docking of LasI-TZD-C8. (**A**) Docking site of LasI with TZD-C8 inhibitor molecule depicted by PyMol molecular viewer with Autodock Vina produced highest energetic ligand pose. (**B**) Specific residue binding of TZD-C8 involves Arg30 and Ile107 hydrogen bonds, embedded in a molecular electrostatic symmetry met [Coulombic-shaded van der Waals surface (red is acidic, blue is basic) and with a grey backbone ribbon] of LasI active site depicted by PyMol viewer. TZD-C8 inhibitor and water molecule are coloured in orange, hydrogen bonds are coloured in turquoise lines.

We constructed an *in silico* model of the interaction between the TZD-C8 ligand and LasI because it had the highest docking score and the most significant biofilm inhibition result. The model was used to identify distinct binding sites. A successful docking of TZD-C8 revealed that binding pocket accessibility for the docking of the TZD-C8 molecule is enabled via a V-shaped binding cleft (Fig. 4A). The V-shaped binding pocket (V-cleft) is positioned as a gap between subunits α-2, α-3, α-4, β-4 and β-5. The LasI active site is constructed as a V-cleft between the parallel β strands β4 and β5 and involves a conserved pocket of amino acids, with Arg23, Phe27 and Trp33 being crucial for SAM binding. The α2 sites bind the SAM and aid in the N-acylation of the C1 position of acyl-ACP^14^. The residues that interact with the ligand TZD-C8 as analysed by PDBsum are as follows: Met1, Ile2, Val26, Arg30, Lys31, Gly32, Trp33, Asp34, Val35, Gln57, Met79, Phe84, Ser103, Arg104, Phe105, Phe117, Val143, Thr144, Thr145, Gly147, Val148, Arg154 and Arg172. Additional protein interface analysis by PISA revealed that the following residues possessed hydrogen binding interaction capabilities with negative ∆G (Gibb’s energy): Arg30, Arg104, Ile107, Val143 and Thr144.

A LIGPLOT view of the interaction schema between LasI and TZD-C8 in the three highest energy binding states showed direct hydrogen bonds between the ligand and Arg30 and Ile107 (Supplementary Data Figure S4A). Consequently, these residues were chosen as SDM targets. Docking analysis between TZD-C8 and LasI revealed hydrophobic interactions in the active site with the conserved LasI residues Val26, Phe27, and Trp33. These residues extend from the α2 helix and play an important role in binding SAM^14^. Arg104 and the other, less energetic ligand states also show some hydrogen bond formation with the residues Phe105, Val143 and Thr144 (Supplementary Data Figure S4B).

## Discussion

### *P. aeruginosa* as a QS inhibition model

The idea of targeting the quorum-sensing pathway (e.g., HSL production) as a potential therapeutic intervention has been previously explored^5^,^39^. However, the full potential of this approach has not yet been realized. This strategy sidesteps conventional mechanisms of antibiotic resistance, as the concept of using a drug to decrease virulence factor production while not affecting growth does not exert evolutionary pressure to force the appearance of resistant strains^40^.

Phenotypic behaviours of *P. aeruginosa*, such as swarming and biofilm formation, are commonly used for testing QS inhibition^39^,^40^,^41^,^42^. There are two types of *P. aeruginosa* biofilm. One is the pellicle (Pel exopolysaccharide dependent) biofilm forming at the air-liquid interface, held by the water surface tension. The other biofilm type is surface-attached. This biofilm is known to be dependent on the Psl exopolysaccharide polymers which attach the biofilm to the surface^21^. The Psl production is controlled by the QS *las* system^43^ so we examined surface biofilm formation to determine the effects of inhibiting QS with TZD. In recent studies, the most obvious choice of QS inhibitors has been signal molecule analogues, such as those involved in LasR activation^39^, or other natural compounds with QS inhibition effects, such as trans-cinnamaldehyde^42^ or cinnamaldehyde^44^, for which *in silico* study approaches have been explored. We chose to study the effect of emerging bacteriostatic thiazolidinedione molecules, as these compounds have been shown to have an effect on the QS *lux* system in *Vibrio harveyi*^16^, and such compounds were so far untested in a similar *P. aeruginosa* model.

One virulence-related phenotype controlled by QS in *P. aeruginosa* is biofilm formation. This phenotype is observed in more than 80% of human infections^40^. The HSL-producing enzyme LasI affects biofilm formation under certain conditions, and the expression of numerous virulence-related genes and virulence-related metabolites^7^,^23^,^45^, making it an ideal target for a non-lethal treatment aimed at decreasing virulence.

Our ability to synthesize different TZD derivatives with variable carbon chain length (C8 or C10) allowed us to manipulate the interactions between the molecules and their potential binding site. Therefore, we were able to perform a structure-function analysis.

The microarray meta-analysis following TZD-C8 exposure revealed a strong correlation with QS inhibition schema. Expression of the *pqs* operon is directly regulated and promoted by the *las* system, therefore downregulation of the entire operon is directly caused by the upstream inhibition effect from the *las* system.

QS Inhibition is also supported by other reported gene downregulation of the expression of *hcnA* and *katA*, which are controlled by the QS system^46^,^47^. The QS and ANR (anaerobic nitrate regulator) mechanisms are intertwined through *katA* and *hcnA* genes. The low activation of ANR regulation can explain the *arcDABC* operon and *dnr* downregulation^47^. Additionally *pqsR* (*mvfR*), a *las* controlled gene, was reported to have differentially regulated *arcA* and *arcD* genes^48^. The expression of *hcnA* is also downregulated as this gene is controlled by the QS regulators of LasR and RhlR^46^.

Downregulation of *pqs* expression is distinguished from the slight upregulation of *kynA* expression. This finding suggests that anthranilic acid, a precursor for PQS molecules^27^, is produced. The meta-data analysis supports a biofilm-reduced phenotype due to the downregulation of both *pqs* operon and *mucA* gene expression. The *pqs* operon is responsible for intact, mature biofilm structures, and *mucA* is responsible for transition to the CF pathogenic mucoid phenotype^38^. PQS is known to stimulate biofilm production^49^.

LasR inhibition could be expressed in a manner similar to LasI inhibition in microarray meta-data. The effect is exerted downstream on the *pqs* operon, in the downregulation of genes such as *hcnA* and *pvdA* and in the minor −0.47-fold change in the expression of *lasI*. The minor *lasI* downregulation could be a result of a negative feedback affect occurring due to a decreased production of 3-oxo-C12-HSL, rather than a LasR downregulation affect^50^, which is supported by our discovery of a direct LasI inhibition (Figs 2 and 4).

### *In vitro* tests reveal QS inhibition

The complex network controlling the QS mechanism in *P. aeruginosa* involves three major operons. The activity of *las*, *rhl*, and *pqs* are required to monitor different pathways to pinpoint the specific target of TZD-C8 inhibition. The specific *las* system (LasI or LasR) inhibited by TZD-C8 is apparent in the wide microarray screen, as transcriptional changes are observed only downstream starting from the *pqs* operon. A β-Gal assay revealed that there was no effect on RhlI, as demonstrated by the lack of change in the production of C4-HSL (Supplementary Data, Figure S4). RhlI and LasI are 31% identical in protein sequence, demonstrating the importance of this RhlI assay. It is well documented in the literature that LasR and RhlR have different structural motifs, suggesting that the different proteins have different inhibitor binding affinities^7^. We have demonstrated a QS inhibition effect that is independent of the expression of the *rhl* system.

Other studies of QS inhibition in *P. aeruginosa* revealed apparent changes in swarming activity due to exposure to anti-QS compounds^42^. The phenotypes exhibited in our *in vitro* tests, such as swarming, were correlated with HSL production levels. While QS inhibition was observed in both point mutated strains of PAO-R30D and PAO-I107S as well as the PAO-JP-comp strain, in reduction of swarming as well as HSL signal production after exposure to the TZD-C8. No change in HSL signal production or swarming was observed between the control and TZD-C8 exposed cultures of the double mutant (Figs 2B and 3A, respectively). The strain PAO1 exhibited the highest reduction in swarming (74%). All the strains which were created as a complementation of PAO-JP1 strain showed significant lower swarming capability although HSL signal reduction showed similar reduction (35–42% [Fig. 3A]).

Only the double mutant PAO-R30D, I107S showed the complete abolishment of QS inhibition (only 4% reduction in HSL production and total 4% increase in swarming), which was what we expected. We presume that the *in vitro* analysis were only sensitive enough to pick up the abolishment of inhibition in the double mutant strain.

The point mutations in LasI did cause some loss of function in LasI signal production (the mutated strains show decreases of 31–37% compared with the PAO-JP-comp control strain). This finding suggests the importance of the SAM binding site residues. Mutations in these residues would have compromised structural integrity, but not completely as the protein still maintains 61–69% of its signal production capability.

### *In silico* modelling predicts the TZD-molecule interaction

*In silico* modeling is an effective tool for understanding the molecular mechanism of interactions and has been used in other studies of QS inhibition^42^,^44^,^50^. AutoDock Vina has been used in prior virtual screens of compounds interacting with proteins, but such compounds were with large molecular weights corresponding to high energy binding. In one example, the compound ‘ZINC4085364’ was found to have a good affinity score with the MurE enzyme of *Mycobacterium tuberculosis*^51^. Our tested compounds have lower molecular weights. This forced us to use a very sensitive *in vitro* test, the QS signal production assay, to find correlations with the docking predictions.

We executed a successful docking analysis with a few control proteins. These proteins were chosen based on their relevance to the *las* system. QscR can bind to LasI and shows a downstream effect on the QS system^52^. The PqsR protein is controlled by the *las* system and controls the transcription of the *pqsABCD* operon and the LasR protein affects the signal received from the LasI protein, all of the proteins mentioned scored a lower affinity in the computer docking analysis than the LasI as a target to the TZD-C8.

In order to understand the mechanisms of TZD-C8 targeting it is important to view the *in silico* and *in vitro* data in the light of other cases of small molecules (TZD or other), which target Lux-type synthases.

It has been documented that the QS system of *Vibrio harveyi* is affected by TZD-C8 and TZD-C10 with the exposure to these molecules decreasing LuxR binding to the DNA and thereby inhibiting transcription activation^16^.

Furthermore, other agents have been documented to have such a dual QS inhibition effect on both *V. harveyi* and *P. aeruginosa*, for example molecules such as the furanone type and cinnamaldehyde type compounds.

Inhibition of the autoinducer production of AI-1 and AI-2 by halogenated-furanone in *V. harveyi* model^53^ and the ‘C-30’ synthetic furanone which inhibited 80% QS controlled genes in *P. aeruginosa* and have shown high specificity to the *las* and *rhl* quorum sensors^54^.

The cinnamaldehyde type molecules inhibited QS in *V. harveyi* and *P. aeruginosa*^42^,^55^, respectively. Specifically, the trans-cinnamaldehyde molecule which showed disruption in *P. aeruginosa* swarming motility as caused by a decrease in HSL signal production of both *las* and *rhl* synthases^42^. Further molecular docking prediction of LasI binding by the trans-cinnamaldehyde was successfully conducted^42^, the LasI binding was in similar localization as the TZD-C8. The trans-cinnamaldehyde committed hydrogen bond with Arg30 and was surrounded by residues of Phe27, Trp33 and Phe105.

Although some homology exist between the *P. aeruginosa las* system to the *Vibrio fischeri*’s *lux* system which has its own acyl-HSL signals^56^, no such similarity exists with the *V. harveyi* hence no explanation so far can say why such overlap in QS mechanisms is demonstrated except a coincidental structural similarity in proteins which produce HSL type molecules.

Another case of LuxI-type synthase, TofI, exhibits a similar mechanism of QS inhibition^57^. The TofI inhibitor of J8-C8 also binds into the catalytic site in similar proximity to residues parallel to those in LasI (Supplementary Data, Figure 7S). The QS modes of inhibition in LuxI-type proteins demonstrated in this paper greatly strengthen our hypothesis of the potential LasI inhibition, in the localization inside the catalytic site and involving the same residues of which are the SAM binding amino acids.

Overall, both the *in silico* calculations and the *in vitro* tests, in this research, indicate that the target of the inhibition by the TZD-C8 is LasI. It cannot be ruled out that other domains in the QS cascade may be affected, directly or indirectly, by the TZD-C8 including a decrease in *V. harveyi* LuxR transcription capability^16^.

Both the LasI and the LasR have similar effects on the *pqs* operon (shown in the microarray results, Table 1S).

While we examine the less energetic ligand interactions predicted by Vina (*in silico* analysis) highlights the involvement of other residues in the inhibition mechanism. These residues could be further tested for a more complete understanding of TZD-C8 binding to LasI by creating a larger pool of SDM strains, in addition to the more favourable energetic residues selected in this study. Overall, the combined approach of utilizing PDBsum and PISA software in the *in silico* analysis achieved good specificity in this residue selection approach.

Our *in vitro* tests using *P. aeruginosa* were only sensitive enough to detect effects in a double mutant. Both Arg30 and Ile107 were selected following the *in silico* prediction. The search for a potential QS inhibitor should be selectively applied to only high-scoring binding inhibitors, as TZD-C8, as done in our study. A site-specific effect of the TZD-C8 molecule emerges through AutoDock Vina calculations (Table S4), which showed an affinity of −6.2 kcal/mol for TZD-C10 and an affinity of −8.1 kcal/mol for TZD-C8. Our *in silico* findings corresponded to our β-Gal plate assay results. The effect due to TZD-C8 was markedly different compared with that of TZD-C10 as treatment with TZD-C10 resulted in no notable change in 3-oxo-C12-HSL signal production.

## Conclusion

Our results show a therapeutic potential for the TZD-C8 agent. TZD-C8 affects *P. aeruginosa* biofilms, probably by inhibiting LasI and the genes that it controls.

We used novel *in silico* predictions complemented by sensitive *in vitro* assays. Direct enzyme and inhibitor interaction experiments are necessary in order to understand the full mechanism of inhibition of TZD derivatives.

*P. aeruginosa* shows a natural tendency to spread by swarming and thrives in a carbon-rich environment. Upon witnessing the ability of TZD molecule to constrain both biofilm formation and swarming, we believe that testing for the *in vivo* effectiveness of these compounds is warranted. A mechanistic model of QS inhibitor interactions is represented by our tested QS inhibitor (TZD-C8). This allows us to propose a molecular scaffold, which can be used to tackle *P. aeruginosa* infections.

## Additional Information

**How to cite this article**: Lidor, O. *et al.* Mechanistic analysis of a synthetic inhibitor of the *Pseudomonas aeruginosa* LasI quorum-sensing signal synthase. *Sci. Rep.* **5**, 16569; doi: 10.1038/srep16569 (2015).

## Supplementary Material

Supplementary Information
