# Corrigendum: Mechanistic analysis of a synthetic inhibitor of the *Pseudomonas aeruginosa* LasI quorum-sensing signal synthase

**DOI:** 10.1038/srep25257

**Published:** 2016-05-09

**Authors:** O. Lidor, A. Al-Quntar, E. C. Pesci, D. Steinberg

Scientific Reports 5: Article number: 16569;10.1038/srep16569 Published online: 11232015; Updated: 05092016

This Article contains typographical errors.

In the Results section under subheading ‘*In silico* evaluation of the interaction between TZD and LasI.’

“A LIGPLOT view of the interaction schema between LasI and TZD-C8 in the three highest energy binding states showed direct hydrogen bonds between the ligand and Arg30 and Ile107 (Supplementary Data Figure S4A)”.

should read:

“A LIGPLOT view of the interaction schema between LasI and TZD-C8 in the three highest energy binding states showed direct hydrogen bonds between the ligand and Arg30 and Ile107 (Supplementary Data Figure S6A).

“Arg104 and the other, less energetic ligand states also show some hydrogen bond formation with the residues Phe105, Val143 and Thr144 (Supplementary Data Figure S4B)”.

should read:

“Arg104 and the other, less energetic ligand states also show some hydrogen bond formation with the residues Phe105, Val143 and Thr144 (Supplementary Data Figure S6B)”.

In the Discussion section under subheading ‘*In silico* modelling predicts the TZD-molecule interaction.’

“The TofI inhibitor of J8-C8 also binds into the catalytic site in similar proximity to residues parallel to those in LasI (Supplementary Data, Figure 7S).

should read:

“The TofI inhibitor of J8-C8 also binds into the catalytic site in similar proximity to residues parallel to those in LasI (Supplementary Data, Figure S7)”.

“Both the LasI and the LasR have similar effects on the pqs operon (shown in the microarray results, Table 1S)”.

should read:

“Both the LasI and the LasR have similar effects on the pqs operon (shown in the microarray results, Table S1)”.

