# Supplementary material for: Mechanistic analysis of a synthetic inhibitor of the Pseudomonas aeruginosa LasI quorum-sensing signal synthase
Source: Sci Rep. 2015 Nov 23;5:16569. doi: 10.1038/srep16569 (PMC4655403; doi:10.1038/srep16569)
Supplement: Supplementary Information [file srep16569-s1.doc]

**Mechanistic analysis of a synthetic inhibitor of the *Pseudomonas aeruginosa* LasI quorum-sensing signal synthase**

O. LIDOR^1*^, A. Al-Quntar^1,2^, E. C. PESCI^3^, D. STEINBERG^1^

^1^Biofilm Research Laboratory, Institute of Dental Sciences, Faculty of Dental Medicine, the Hebrew University of Jerusalem.

^2^Institute of Drug Research, School of Pharmacy, The Hebrew University of Jerusalem,

^3^Department of Microbiology and Immunology, Brody School of Medicine, East Carolina University, U.S.A.

*Corresponding author

E-mail: ofir.lidor@mail.huji.ac.il

**Supplementary Tables**

Table S1: Bacterial strains and plasmids

| ***P. aeruginosa* strains** | | |
| --- | --- | --- |
| **PAO1** | Wild-type | [1, 2] |
| **PDO100** | *rhlI* : :Tn501-2; derived from strain PAO1 | [3, 6] |
| **PAO-JP1** | *lasI* : :Tet; derived from strain PAO1 | [2, 3, 4 and 6] |
| **PAO-JP2** | *lasI* : :Tet, *rhlI* : :Tn501-2; derived from strain PDO100 | [2, 3, 4 and 6] |
| **PAO-QA1** | *pqsA* deletion mutant derived from PAO1 strain | [4] |
| **PAO-JP-comp** | PAO-JP (∆*lasI*) contains pJP-comp plasmid | In this article |
| **MG4 (pKDT17)** | *E.coli* 3-oxo-C12-HSL bioassay | [5] |
| **DH5α (pECP61.5)** | *E.coli* C4-HSL bioassay | [6] |
| **PAO-R30D** | PAO-JP (∆*lasI*) contains pJP-comp plasmid: R30D substitution | In this article |
| **PAO-I107S** | PAO-JP (∆*lasI*) contains pJP-comp plasmid: I107S substitution | In this article |
| **PAO-R30D, I107S** | PAO-JP (∆*lasI*) contains pJP-comp plasmid: R30D +I107S substitutions. | In this article |
| **Plasmids** | | |
| **pJP1-comp** | pEX1.8 complement expression plasmid with an insert of *lasI* sequence. | In this article |
| **pEX1.8** | *E. coli-P. aeruginosa* shuttle plasmid containing inducible IPTG *tac* promoter. | [4, 6] |
| **pMRP9-1** | Crb : GFP protein expressing plasmid under *tac* promoter | [7] |

**Table S2**: **Known Genes which showed a significant difference in expression (P<<0.01).** Exposure of strain PAO1 to 0.02 mM of TZD-C8. Standard criteria for analysis include: Adjusted P. value less than 0.05, Average Expression of signal is larger than 9.0, B value is greater than 1.5 and

0.3< logFC < -0.3.

| **Locus number^a^** | **Description** | **Gene symbol** | **M^b^** | **P Value^c^** | **B^d^** |
| --- | --- | --- | --- | --- | --- |
| **PA5171** | arginine deiminase | arcA | -2.28132 | 5.40E-23 | 41.58889 |
| **PA5170** | arginine/ornithine antiporter | arcD | -2.0886 | 1.56E-19 | 34.41665 |
| **PA5172** | ornithine carbamoyltransferase, catabolic | arcB | -2.04423 | 2.69E-16 | 27.29368 |
| **PA5173** | carbamate kinase | arcC | -1.98908 | 2.06E-18 | 31.98538 |
| **PA4067** | Outer membrane protein OprG precursor | oprG | -1.60163 | 5.57E-16 | 26.58204 |
| **PA5427** | alcohol dehydrogenase | adhA | -1.33688 | 1.46E-11 | 16.47258 |
| **PA1546** | coproporphyrinogen III oxidase | hemN | -1.2249 | 1.63E-11 | 16.35967 |
| **PA4587** | cytochrome c551 peroxidase precursor | ccpR | -1.17484 | 3.13E-11 | 15.70606 |
| **PA2193** | hydrogen cyanide synthase HcnA | hcnA | -1.15397 | 7.89E-15 | 23.97546 |
| **PA0998** | beta-keto-acyl-acyl-carrier protein synthase-like protein | pqsC | -1.08177 | 4.50E-15 | 24.52825 |
| **PA1000** | Quinolone signal response protein | pqsE | -0.95603 | 1.80E-12 | 18.57264 |
| **PA0997** | beta-keto-acyl-acyl-carrier protein synthase-like protein | pqsB | -0.93152 | 1.41E-13 | 21.112 |
| **PA3531** | Bacterioferritin | bfrB | -0.89974 | 8.27E-14 | 21.64637 |
| **PA0999** | 3-oxoacyl-[acyl-carrier-protein] synthase III | pqsD | -0.79838 | 1.73E-12 | 18.61341 |
| **PA0996** | probable coenzyme A ligase | pqsA | -0.78603 | 8.02E-11 | 14.75874 |
| **PA4922** | azurin precursor | azu | -0.7417 | 2.32E-05 | 2.103496 |
| **PA4235** | Bacterioferritin | bfrA | -0.5991 | 2.29E-10 | 13.70368 |
| **PA4762** | heat shock protein GrpE | grpE | -0.585 | 1.37E-05 | 2.627259 |
| **PA0024** | coproporphyrinogen III oxidase | hemF | -0.48596 | 8.88E-08 | 7.691389 |
| **PA1432** | autoinducer synthesis protein LasI | lasI | -0.47812 | 6.59E-09 | 10.35171 |
| **PA4566** | GTP-binding protein Obg | obg | -0.47261 | 1.73E-05 | 2.3953 |
| **PA1561** | aerotaxis receptor Aer | aer | -0.46723 | 2.18E-07 | 6.787852 |
| **PA4236** | Catalase | katA | -0.46675 | 1.57E-05 | 2.496512 |
| **PA4542** | ClpB protein | clpB | -0.44608 | 1.39E-05 | 2.612651 |
| **PA0852** | chitin-binding protein CbpD precursor | cbpD | -0.39093 | 8.16E-06 | 3.148127 |
| **PA4565** | gamma-glutamyl kinase | proB | -0.35067 | 1.04E-06 | 5.21785 |
| **PA1049** | pyridoxamine 5'-phosphate oxidase | pdxH | -0.32558 | 6.10E-07 | 5.751131 |
| **PA4759** | dihydrodipicolinate reductase | dapB | -0.30352 | 1.37E-07 | 7.25083 |
| **PA5495** | homoserine kinase | thrB | -0.28347 | 1.23E-05 | 2.741313 |
| **PA0763** | anti-sigma factor MucA | mucA | -0.2674 | 2.00E-05 | 2.254271 |
| **PA4545** | competence protein ComL | comL | 0.243766 | 2.53E-05 | 2.018728 |
| **PA3013** | acetyl-CoA acetyltransferase | foaB | 0.333022 | 4.12E-06 | 3.831541 |
| **PA3014** | fatty-acid oxidation complex alpha-subunit | faoA | 0.366858 | 8.72E-06 | 3.082375 |
| **PA2579** | hypothetical protein | kynA | 0.376344 | 2.30E-07 | 6.73039 |
| **PA4228** | pyochelin biosynthesis protein PchD | pchD | 0.390238 | 1.92E-05 | 2.295504 |
| **PA2248** | 2-oxoisovalerate dehydrogenase (beta subunit) | bkdA2 | 0.398417 | 7.79E-06 | 3.194884 |
| **PA4221** | Fe(III)-pyochelin outer membrane receptor precursor | fptA | 0.435204 | 5.96E-06 | 3.462879 |
| **PA0672** | heme oxygenase | hemO | 0.463338 | 2.19E-06 | 4.467546 |
| **PA0500** | biotin synthase | bioB | 0.464331 | 3.28E-08 | 8.696279 |
| **PA0265** | succinate-semialdehyde dehydrogenase | gabD | 0.469177 | 4.64E-09 | 10.66808 |
| **PA4370** | Insulin-cleaving metalloproteinase outer membrane protein precursor | icmP | 0.525284 | 1.18E-11 | 16.68473 |
| **PA0266** | 4-aminobutyrate aminotransferase | gabT | 0.556909 | 5.57E-09 | 10.48386 |
| **PA5531** | TonB protein | tonB | 0.586195 | 1.73E-11 | 16.3038 |
| **PA2247** | 2-oxoisovalerate dehydrogenase (alpha subunit) | bkdA1 | 0.629656 | 8.81E-07 | 5.380673 |
| **PA2386** | L-ornithine N5-oxygenase | pvdA | 0.633542 | 3.37E-07 | 6.34681 |
| **PA2015** | Citronelloyl-CoA dehydrogenase, GnyD | gnyD | 0.699566 | 4.86E-08 | 8.299082 |
| **PA0424** | multidrug resistance operon repressor MexR | mexR | 0.890206 | 6.91E-13 | 19.52957 |
| **PA0427** | Major intrinsic multiple antibiotic resistance efflux outer membrane protein OprM precursor | oprM | 1.00139 | 1.76E-15 | 25.46484 |
| **PA0865** | 4-hydroxyphenylpyruvate dioxygenase | hpd | 1.097663 | 3.99E-12 | 17.77395 |
| **PA0425** | Resistance-Nodulation-Cell Division (RND) multidrug efflux membrane fusion protein MexA precursor | mexA | 1.22068 | 3.98E-12 | 17.77593 |
| **PA0426** | Resistance-Nodulation-Cell Division (RND) multidrug efflux transporter MexB | mexB | 1.226787 | 3.48E-17 | 29.27779 |

^a^ Based on the genome annotation of *P. aeruginosa* provided by TIGR.

^b^ Log2-fold expression according to the M value, M > 0 means upregulation and M < 0 downregulation of the gene, M = fold change.

^c^ Moderated *t*-test and its corresponding P value, adjusted by Benjamini and Yekutiely method.

^d^ Bayesian test value, meaning the probability for a gene to be differentially expressed. Genes are listed according to their decreasing statistical importance according to their B-value.

**Table S3. List of primers used in this study**

| **cloning pJP-comp plasmid** | Reverse:  Forward: | 5'- ATGATCGTACAAATTGGTCGGC-3'  5'-AAAAAAGCTTTTTACAGCGGATTCGGCA-3' |
| --- | --- | --- |
| **Cloning *lasI*- I107S** | Reverse:  Forward: | 5'-CTCAGCCGTTTCGCCAGCAACTCTGGACAGAAA-3'  5'-TTTCTGTCCAGAGTTGTGGCGAAACGGCTGAG-3' |
| **cloning *lasI*- R30D** | Reverse:  Forward: | 5'-CTCAAGTGTTCAAGGAGGACAAAGGCTGGGACGTTAG-3'  5'- CTAACGTCCCAGCCTTTGTCCTCCTTGAACACTTGAGC- 3' |

| **QscR** | **PqsR** | **LasR** | **LasI** | **compound** |
| --- | --- | --- | --- | --- |
| -4.5 | -4.3 | -4.3 | -4.2 | TZD |
| -5.3 | -4.8 | -7 | -8.1 | TZD-C8 |
| -6.5 | -5.7 | -5 | -6.2 | TZD-C10 |

**Table S4. *In-silico* prediction performed by Autodock Vina software.** Affinity calculation (kcal/mol) of LasI, PpqsR, QscR and LasR proteins to the different TZD molecules, in rigid structure (pdb file origin is detailed in *In silico* part of materials & methods). The calculation chosen for the graph represents only of the highest energetic ligand configuration (with 0.0 rmsd).

**Supplementary Figures**


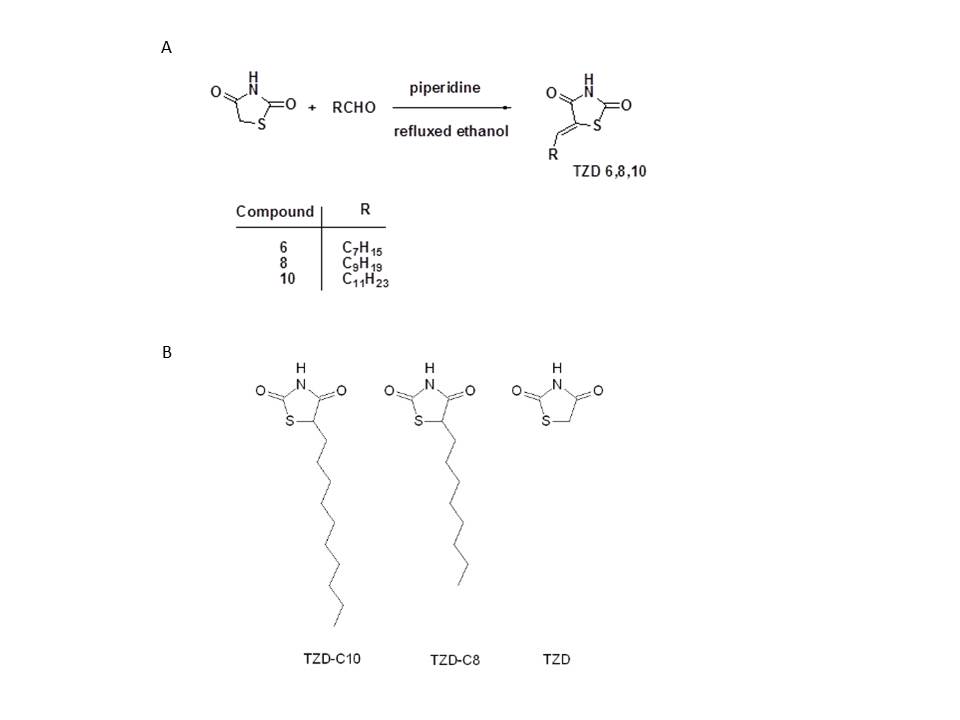


Figure S1. TZD synthesis pathway. (A) Thiazolidinedione derivatives with a diffrent R side carbon chain. TZD-C8 ((*z*)-5-octylidenethiazolidine-2, 4-dione) and TZD-C10 ((*z*)-5-decylidenethiazolidine-2, 4-dione) synthesis.

(B) TZD derivatives chemical structure used in this study; TZD, TZD-C8, TZD-C10.


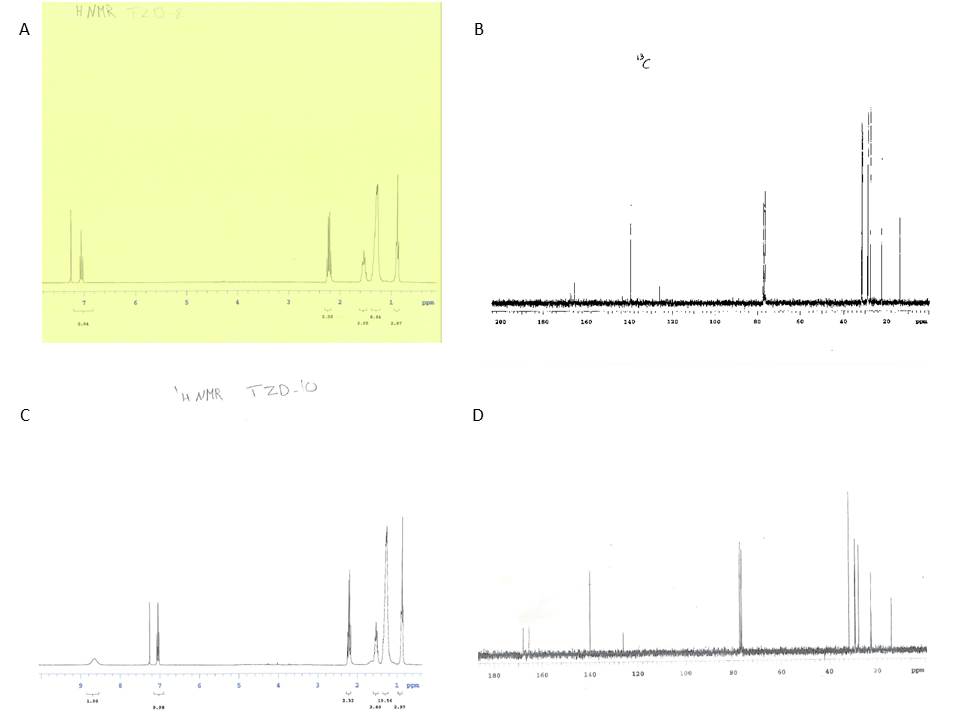


**Figure** **S2 and ^13^C-NMR** analysis of**: A**)^1^H-NMR of TZD-C8 , **B**) ^13^C-NMR of TZD-C8**,** **C**)^1^H-NMR of TZD-C10 , **D**) ^13^C-NMR of TZD-C10**.**

NMR analysis of TZD-8:

Melting point: 71 ^0^C; ^1^H NMR (300 MHz, Chloroform d): δ 0.85 (t, 3H, *J*_HH_ = 7.2 Hz), 1.20-1.40 (overlap, 8H), 1.57 (m, 2H), 2.21 (m, 2H), 7.07 (t, 1H, *J*_HH_ = 8.4 Hz), 8.63 (broad s, 1H); ^13^C NMR (75.5 MHz, Chloroform d): δ14.07, 22.56, 27.78, 28.91, 29.12, 31.62, 31.82, 126.18, 139.88, 165.80, 167.50; Anal. Calcd for C_11_H_17_NO_2_S: C, 58.12; H, 7.54; N, 6.16; S, 14.11. Found: C, 57.91; H, 7.47; N, 6.27; S, 14.29.

NMR analysis of TZD-10:

Melting point: 64 ^0^C; ^1^H NMR (300 MHz, Chloroform d): δ 0.88 (t, 3H, *J*_HH_ = 6.3 Hz), 1.15-1.41 (overlap, 12H), 1.54 (m, 2H), 2.22 (m, 2H), 7.05 (t, 1H, *J*_HH_ = 7.8 Hz), 8.66 (broad s, 1H); ^13^C NMR (75.5 MHz, Chloroform d): δ14.10, 22.66, 27.81, 29.20, 29.24, 29.27, 29.41, 31.84, 126.16, 139.90, 165.55, 168.05; Anal. Calcd for C_13_H_21_NO_2_S: C, 61.14; H, 8.29; N, 5.48; S, 12.56. Found: C, 60.96; H, 8.16; N, 5.62; S, 12.71.


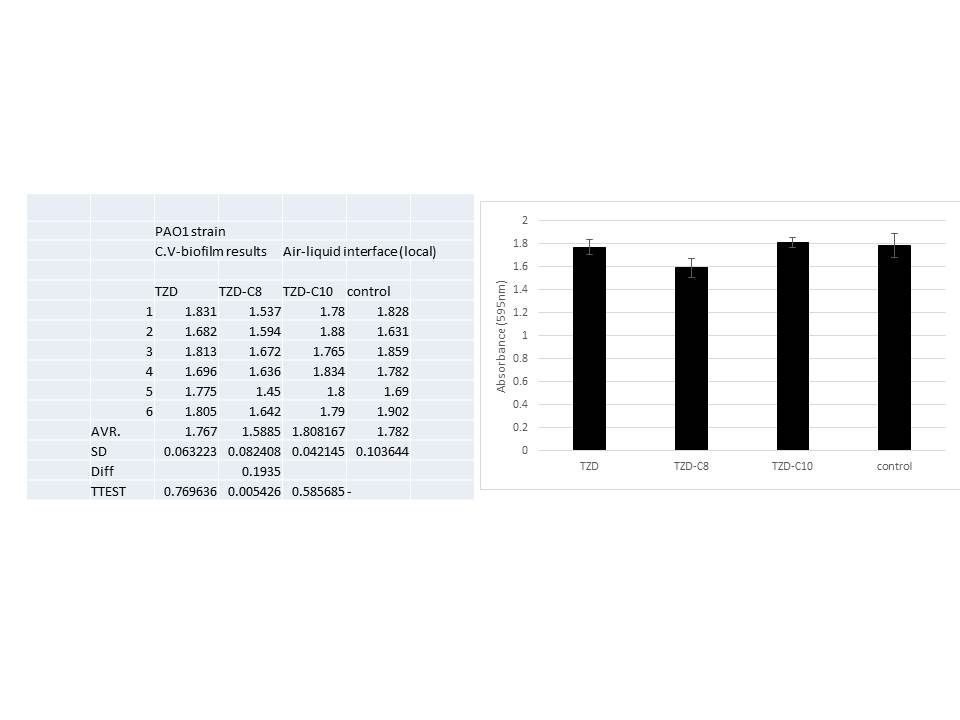


**Figure S3**. **Crystal Violet staining of static *P. aeruginosa* biofilms**; total biofilm of *P. aeruginosa* (air-liquid interface at the sides of micro-well) measured via crystal violet protocol at absorbance (595nm), after exposure to TZD compounds (at final concentration of 0.02 mM).

The bottoms of the wells were cleared with ethanol 95% prior to CV analysis of the pellicle biofilm. SD error bars are assigned. No distinct change was apparent in pellicle size (air-liquide biofilm); data validation was done according to the statistical analysis part (materials & methods).

Figure S4. C4-HSL reporting by the DH5α (pECP61.5) bioassay strain, comparriosn of *P. aerugonosa* PAO1 (wild type strain) TZD-C8 (0.02 mM) exposed Vrs. Non-exposed.

Detection was applied in a standard Miller units analysis approach as in other studies (5, 6). SD error bars are assigned. Data validation was done according to the statistical analysis part (materials & methods). Conclusion: No apparent change is evident between the 2 groups.

**Figure S5. The minimal concentration of TZD-C8 which effects growth of *P. aeruginosa* bacteria**, kinetic measurements in optical density 595nm (OD595).

Conclusion: No apparent effect is evident in bacterial growth between the highest used TZD-C8 concentration (0.02 mM) and the control.


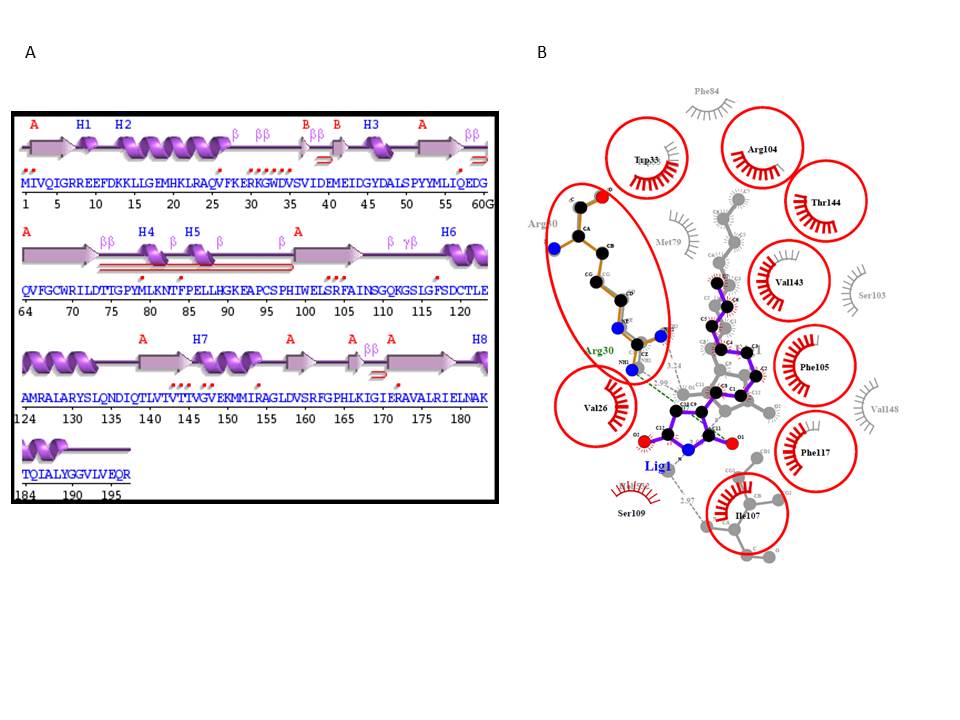


**Figure S6.** (**A**) Ligand binding residues depicted by PDBsum. Residues considered in interaction with the TZD-C8 ligand are marked with red squares. (**B**) LIGPLOT (PDBsum) 2D view of interacting residues of LasI with TZD-C8 inhibitor, constructed with the 3 highest energetic ligand configurations. Red circles indicate residues considered in interaction (LasI and TZD-C8) in more than one configuration.


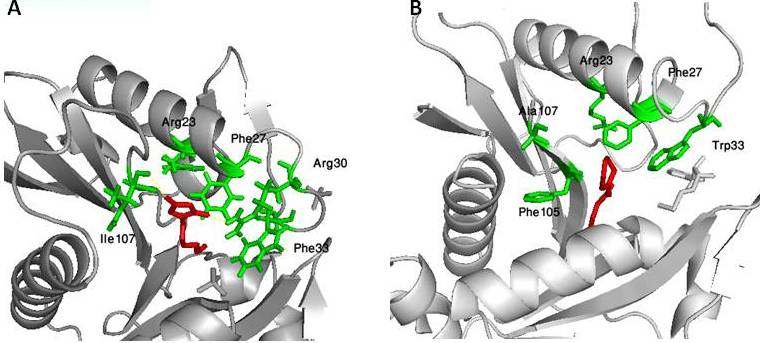


**Figure S7**. Visualized representation of LuxI type protein interaction with inhibitor; (**A**) LasI with TZD-C8 complex, composed after *in-silico* docking (this research). Specific predicted interacting residues (in green) and located inhibitor (in red). (**B**) TofI protein, a *Burkholderia glumae* LuxI-type protein with 36% similarity to LasI (taken from 3P2H pdb data file), in complex with J8-C8 inhibitor molecule (8). Similar located residues in TofI (in green) and located inhibitor (in red).

**Additional Information:**

1) **PDBsum** passwords for case no. k032, i772 and k031 are as follows: 100647, 102402 and 103639.

2) **SDM (site-directed mutagenesis) analysis**:

SDM *P. aeruginosa* mutants sequencing analysis (sequence scanner 2, applied biosystems):

PAO-I107S:


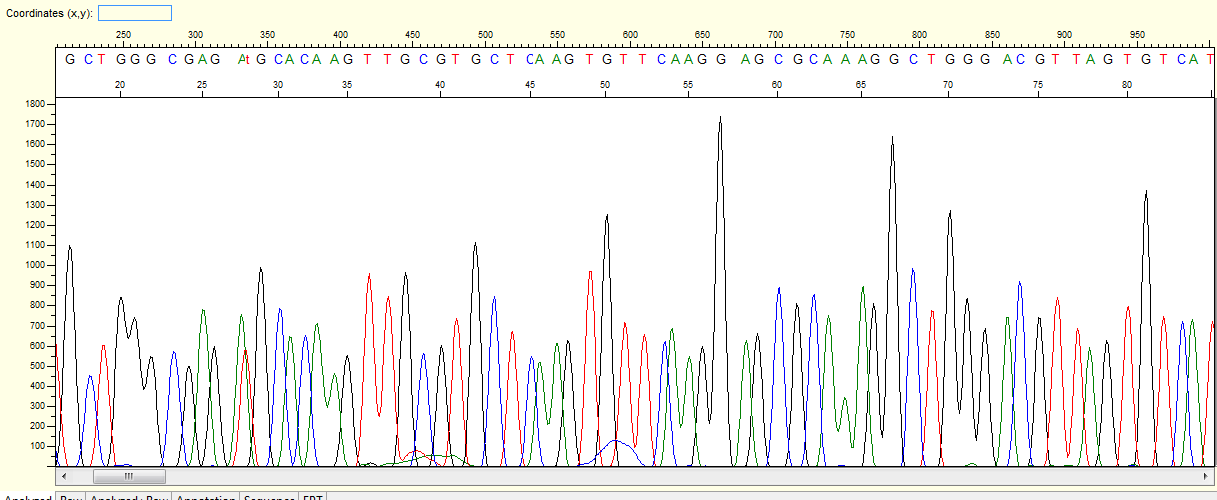


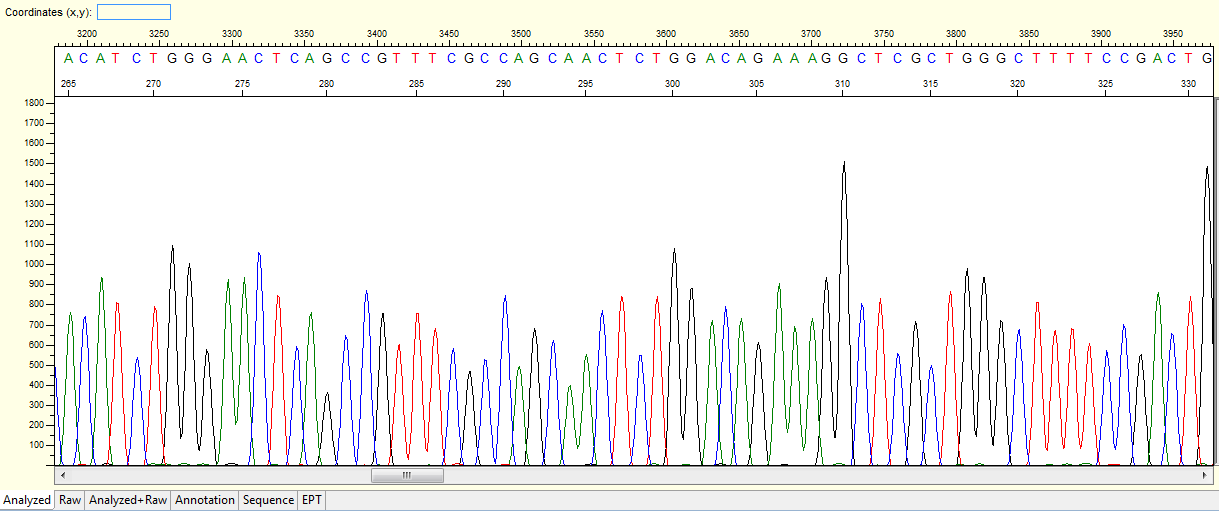


Mutation: ATC (Ile)🡪AGC (Ser), coordinate X= 3510.

PAO-R30D:


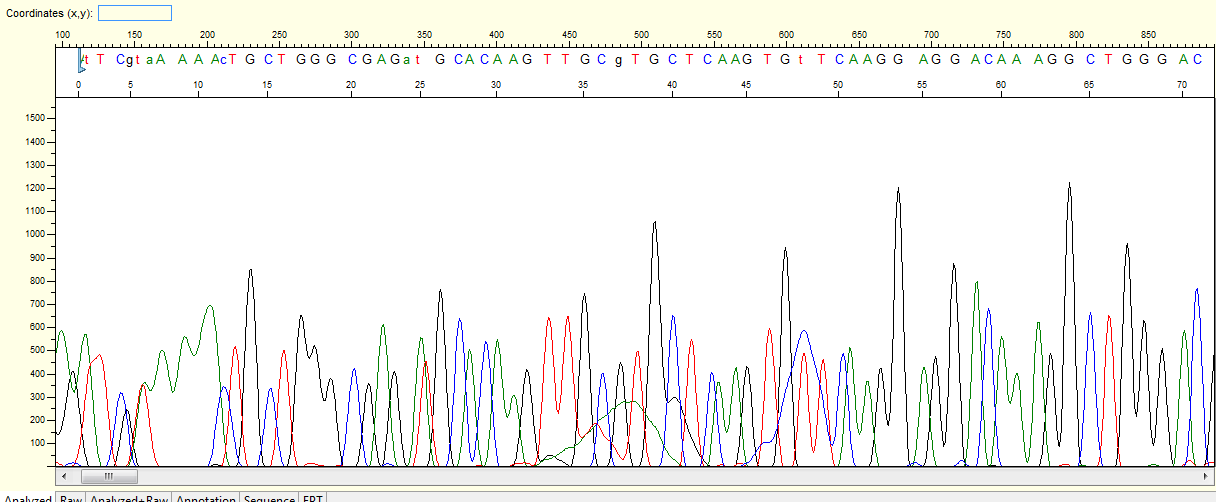


Mutation: CGC (Arg)🡪GAC (Asp), coordinate X= 720.

PAO-R30D, I107S:


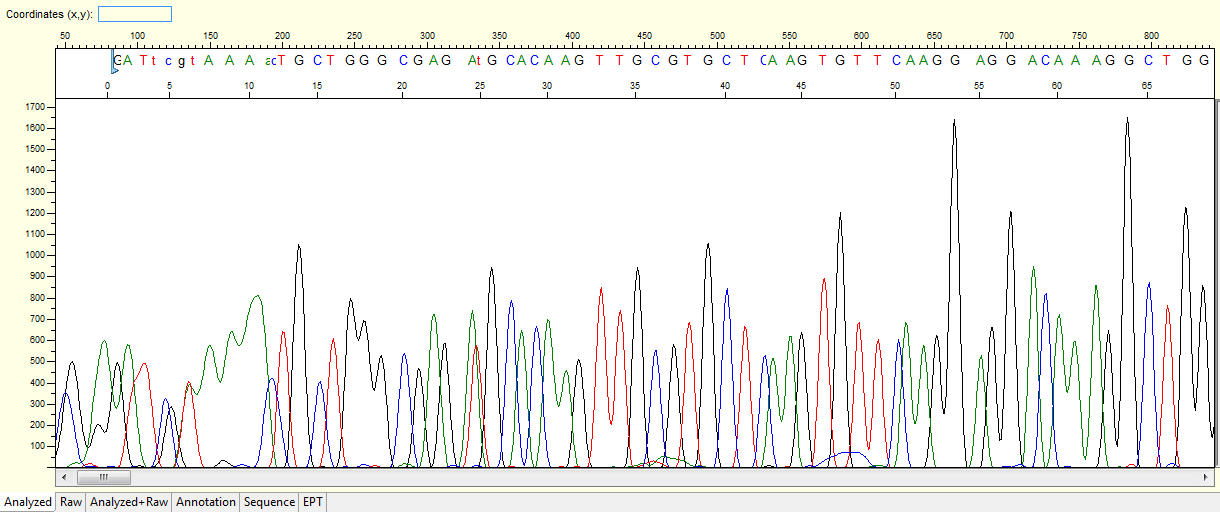


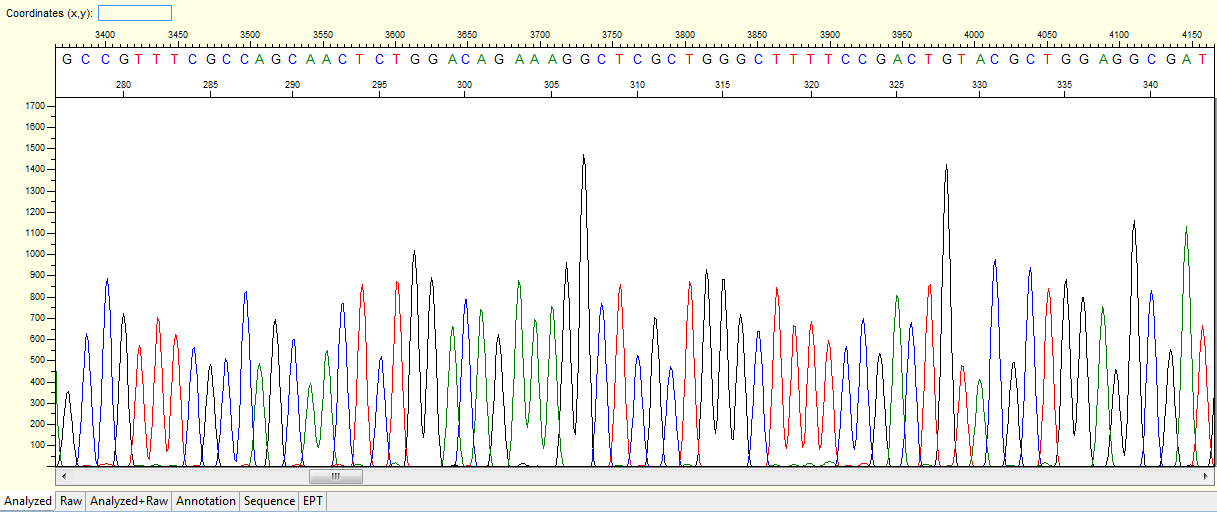


3) **β-Gal modified plate assay**

Biosensors are a viable tool to assay bacterial cultures for quorum sensing (QS) activity, the QS signals are extracted from *Pseudomonas aeruginosa* bacteria using organic solvents and identified on an agar plate overlaid with the biosensor strain.

A modified assay which its purpose is to apply a wide screening technique to quantify the quorum sensing (QS) signals produced by the *Pseudomonas aeruginosa* bacteria. The assay is designed for the *las* signaling pathway, but can be applied to any other β-galactosidase enzyme reporting system.

The Homo-serine lactone signals produced by the *Pseudomonas* bacteria divide into 2 groups: the *N*-butanoyl-homoserine lactone (C4-HSL) a product of the QS *rhl* system and the *N*-(3-oxododecanoyl)-_L_-homoserine lactone(3-oxo-C12-HSL), the latter is the product of the *las* QS system. The *las* system is composed of the signal producing synthase (LasI) and the receptor protein (LasR) which binds the signal and by doing so activates a transcription pathway downstream and positively regulates both the *rhl* and the *pqs* systems. For a review of the QS system see Williams and Càmara (9).

Our system is composed of an *E. coli* bioassay strain carrying both the *lasB-lacZ* translational fusion and *tacp-lasR* under the control of the *lac* promoter (5), the bioassay receives the 3-oxo-C12-HSL signal through the LasR and binds to the *lasB* segment which then transcribing downstream the *lacZ* reporting gene.

The bioassay detection was further modified using an agar plating technique based on the Golberg et al method (10) in order to screen a wide range of bacterial extracts.

**Method protocol**

The modified plate assay procedure is shown in **figure 1#.**

1. The *E. coli* biosensor of MG4 (pKDT17), described in the paper, is plated on a LB agar plate and grown overnight in 37°C.
2. Inoculate a single colony of the biosensor into a fresh 5 ml of LB medium and grown overnight at 37°C and 200 RPM.
3. Autoclave a fresh LB agar medium and allow to cool to 40°C, supplement the media with 80 μg/ml of 5-bromo-4-chloro-3-indolyl-β-D-galactopyranoside (X-gal).
4. Mix a 45 ml of the LB agar medium (cooled to 40°C) with the 5 ml of the biosensor overnight culture. And pour 25 ml of the mix onto a clean Petri dish.
5. After the agar is solidified place paper disks containing bacteria extracts (5 µl of extract in each disk). The extraction process is described in the paper.
6. Incubate the plates in a 30°C incubator overnight to allow the blue zone to develop.
7. Cut the blue zone aseptically and melt with 1 ml of silica "glass milk" solution (MP, Solon, OH, USA). Centrifuge the bacterial debris at 12000 RPM for 2 min and extract with a pipet 800 µl into a clean cuvette.
8. Measure the absorbance at 650 nm.


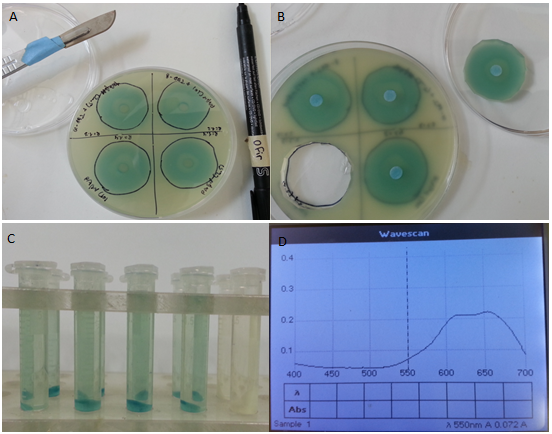


**Figure 1#. Procedure of β-galactosidase blue zone extraction and quantification. A. Marking the blue zone under the petri dish. B. cutting the blue zone. C. melting the agar and centrifuge; the far tube on the right represent the *control. D. Scanning absorbance wave lengths to discover a peak at the 650 nm (repeatedly depicted). *the control tube is composed of a bioassay plated without exposure to the bacterial extracts (paper disks).**

**Calibration curve**

The bioassay exposed to rising concentration of synthetic 3-oxo-C12-HSL (sigma) were used to calibrate the absorbance range of the bacterial extracts, each measurement of β-galactosidase product depicted at 650 nm after absorbance wave scan was done by the GeneQuant 1300 spectrophotometer (GE Healthcare, Uppsala, Sweden**),**  (**figure 2#**).


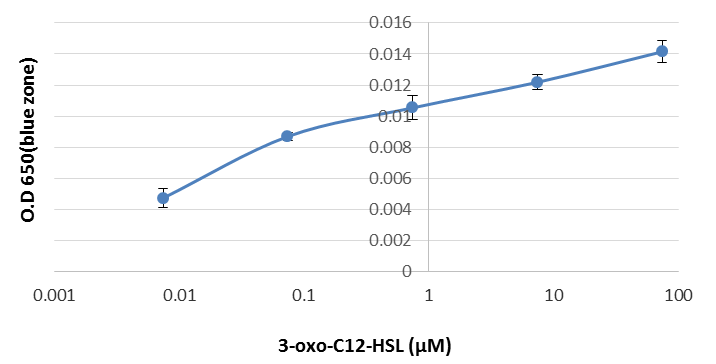


**Figure 2#. Calibration curve of 3-oxo-C12-HSL reported by the bioassay after exposure to synthetic signals (sigma). Each spot represents a triplicate of experiments (6 counts in total). The results are calculated as blue color enumeration.**

A clear logarithmic graph was depicted, enabling us to relatively quantify at 0.1-100 µM of 3-oxo-C12-HSL concentrations. This method allowed us to evaluate the relative count of the *las* signals with a wide screen of extracts.

4) **RNA Validation via BioAnalyzer** (pictures):

*BioAnalyzer RNA analysis: samples used for microarray-are numbered as 1 to 8.

**
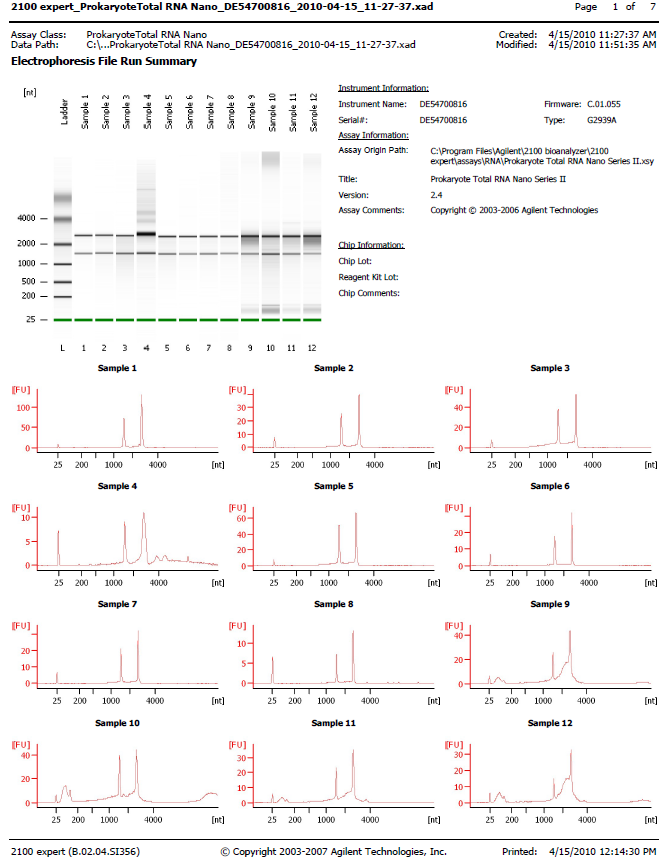
**

**
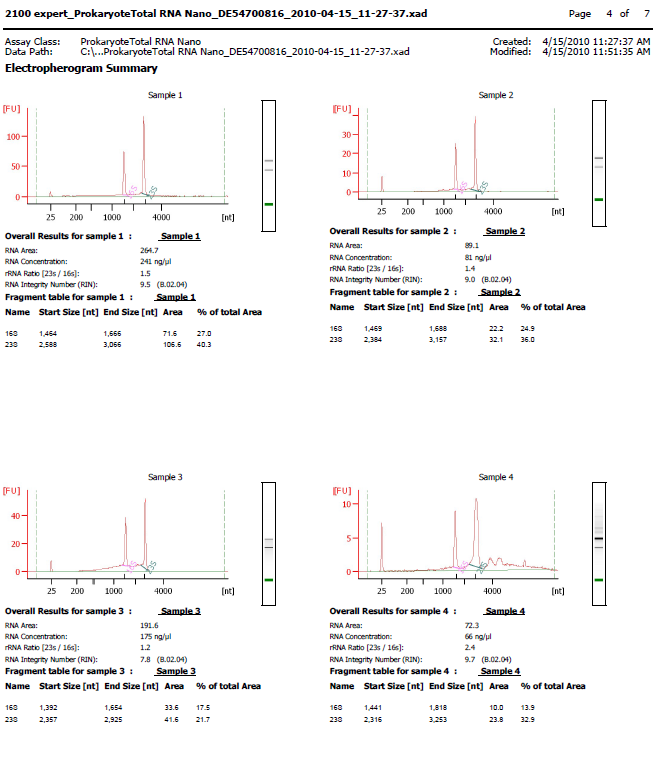
**

**
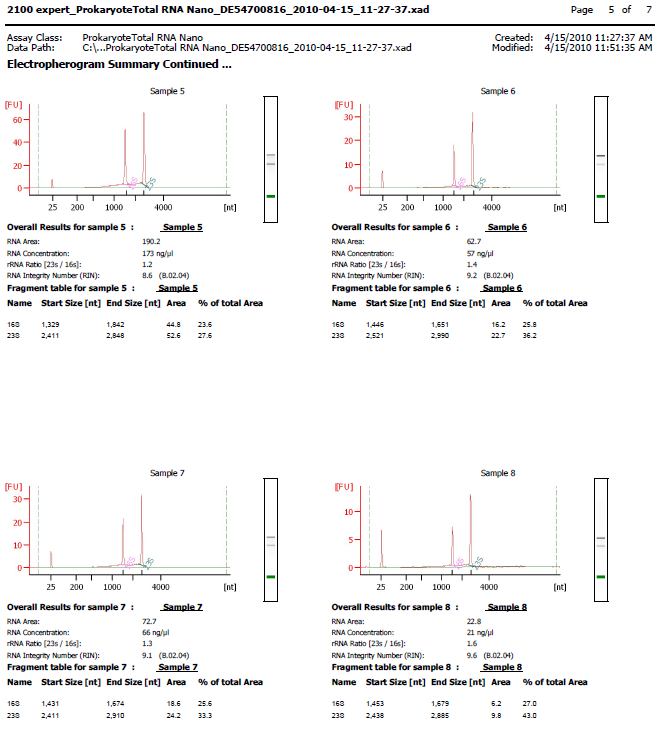
**

**References:**

1. Pesci, E. C. et al. Quinolone signaling in the cell-to-cell communication system of Pseudomonas aeruginosa. *Proc Natl Acad Sci U S A*. **96**, 11229-11234 (1999).
2. Holloway, B. W., Krishnapillai, V. & Morgan, A. F. Chromosomal genetics of Pseudomonas. *Microbiol. Rev.* **43**, 73-102 (1979).
3. McGrath, S., Wade, D. S., Pesci, E. C. Dueling quorum sensing systems in Pseudomonas aeruginosa control the production of the Pseudomonas quinolone signal (PQS). *FEMS Microbiol Lett*. **230**, 27-34 (2004).
4. Farrow, J. M. 3^rd^, et al. PqsE functions independently of PqsR-Pseudomonas quinolone signal and enhances the rhl quorum-sensing system. *J Bacteriol*. **190**, 7043-7051 (2008).
5. Pearson, J. P., Gray, K. M., Passador, L., Tucker, K. D., Eberhard, A., Iglewski, B. H., Greenberg, E. P. Structure of the autoinducer required for expression of Pseudomonas aeruginosa virulence genes. *Proc Natl Acad Sci U S A*. **91**: 197-201 (1994).
6. Pearson, J. P., Pesci, E. C. & Iglewski, B. H. Roles of Pseudomonas aeruginosa las and rhl quorum sensing systems in control of elastase and rhamnolipid biosynthesis genes. *J Bacteriol*. **179**, 5756-5767 (1997).
7. Banin, E., Vasil, M. L., Greenberg, E. P. Iron and Pseudomonas aeruginosa biofilm formation. *Proc Natl Acad Sci* *U S A*. **102**, 11076-11081. (2005).
8. Chung J. et al. Small-molecule inhibitor binding to an N-acyl-homoserine lactone synthase. *Proc Natl Acad Sci U S A*. **108**, 12089-12094 (2011).
9. Williams P., Cámara M. Quorum sensing and environmental adaptation in Pseudomonas aeruginosa: a tale of regulatory networks and multifunctional signal molecules. *Curr Opin Microbiol*. **12,** 182-191 (2009).
10. Golberg, K., Eltzov, E., Shnit-Orland, M., Marks, R. S., Kushmaro, A. Characterization of quorum sensing signals in coral-associated bacteria. *Microb Ecol*. **61**, 783-792 (2011).
